# Bladder cancer in the EMPA-REG OUTCOME trial

**DOI:** 10.1007/s00125-017-4430-0

**Published:** 2017-09-14

**Authors:** Sven Kohler, Jisoo Lee, Jyothis T. George, Silvio E. Inzucchi, Bernard Zinman

**Affiliations:** 1 Boehringer Ingelheim Pharma GmbH & Co. KG, Binger Strasse 173, 55216 Ingelheim, Germany; 2 Section of Endocrinology, Yale University School of Medicine, New Haven, CT USA; 3 Lunenfeld-Tanenbaum Research Institute, Mount Sinai Hospital, University of Toronto, Toronto, Canada

**Keywords:** Bladder cancer, Cancer, Empagliflozin, SGLT2 inhibitors, Type 2 diabetes

*To the Editor:* We read with interest the recently published article in *Diabetologia* by Tang et al on the risk of cancer in patients with type 2 diabetes treated with sodium–glucose cotransporter 2 (SGLT2) inhibitors [1]. We agree with their conclusion that treatment with SGLT2 inhibitors is not associated with a significantly increased risk of cancer. In their analysis of specific cancer types, the authors suggest that SGLT2 inhibitors may be associated with an increased risk of bladder cancer. With respect to empagliflozin, the authors report that the cardiovascular outcomes trial, EMPA-REG OUTCOME, conducted in individuals with type 2 diabetes and established cardiovascular disease, contributed over 50% of individuals and events to their analysis of bladder cancer. We would like to draw attention to data from this trial that were not considered by the authors, which (except for the data on the events reported up to 7 days after the last intake of study drug) were included in documents submitted to the US Food and Drug Administration [2]. In the EMPA-REG OUTCOME trial, cases of bladder cancer were identified from adverse events coded using the following preferred terms in the Medical Dictionary for Regulatory Activities (MedDRA): ‘bladder cancer’; ‘bladder cancer recurrent’; ‘bladder cancer transitional cell carcinoma’; and ‘transitional cell carcinoma’. Transitional cell carcinoma accounts for over 90% of all bladder cancers [3]. Documentation relating to events coded as transitional cell carcinoma was medically reviewed and cases confirmed. In an on-treatment analysis based on events reported from the first intake of study drug up to 7 days after the last intake, four transitional cell carcinoma cases were located in the bladder (three in the placebo group, one in the empagliflozin 10 mg group) and one was located in the ureter (in the empagliflozin 25 mg group). In an intent-to-treat analysis (based on events reported from the first intake of study drug up to trial termination in patients treated with ≥ 1 dose of study drug), bladder cancer was reported in 5/2333 patients (0.2%) in the placebo group, 3/2345 patients (0.1%) in the empagliflozin 10 mg group and 9/2342 patients (0.4%) in the empagliflozin 25 mg group (Table 1).

**Table 1:** Bladder cancer events in the EMPA-REG OUTCOME trial

Events	Placebo	Empagliflozin 10 mg	Empagliflozin 25 mg	Empagliflozin pooled
Events from first intake of study drug up to 7 days after the last intake of study drug	*n* = 2333	*n* = 2345	*n* = 2342	*n* = 4687
Bladder cancer	3 (0.1)	3 (0.1)	8 (0.3)	11 (0.2)
Bladder cancer	0	1 (< 0.1)	5 (0.2)	6 (0.1)
Bladder cancer recurrent	0	0	1 (< 0.1)	1 (< 0.1)
Bladder cancer transitional cell carcinoma	0	1 (< 0.1)	1 (< 0.1)	2 (< 0.1)
Transitional cell carcinoma	3 (0.1)	1 (< 0.1)	1 (< 0.1)	2 (< 0.1)
Events from first intake of study drug up to trial termination	*n* = 2333	*n* = 2345	*n* = 2342	*n* = 4687
Bladder cancer	5 (0.2)	3 (0.1)	9 (0.4)	12 (0.3)
Bladder cancer	1 (< 0.1)	1 (< 0.1)	6 (0.3)	7 (0.1)
Bladder cancer recurrent	0	0	1 (< 0.1)	1 (< 0.1)
Bladder cancer transitional cell carcinoma	0	1 (< 0.1)	1 (< 0.1)	2 (< 0.1)
Transitional cell carcinoma	4 (0.1)	1 (< 0.1)	1 (< 0.1)	2 (< 0.1)
Events with onset after 6 months’ cumulative exposure up to trial termination	*n* = 2187	*n* = 2216	*n* = 2190	*n* = 4406
Bladder cancer	4 (0.2)	3 (0.1)	7 (0.3)	10 (0.2)
Bladder cancer	1 (< 0.1)	1 (< 0.1)	5 (0.2)	6 (0.1)
Bladder cancer recurrent	0	0	0	0
Bladder cancer transitional cell carcinoma	0	1 (< 0.1)	1 (< 0.1)	2 (< 0.1)
Transitional cell carcinoma	3 (0.1)	1 (< 0.1)	1 (< 0.1)	2 (< 0.1)

Data are *n* (%) with events in patients treated with ≥1 dose of study drug

Bladder cancer events were identified based on Medical Dictionary for Regulatory Activities (MedDRA) preferred terms (plus medical review of cases for events coded as transitional cell carcinoma)

To elucidate the potential association between a substance and carcinogenesis, length of exposure needs to be considered. We undertook an additional analysis of patients with at least 6 months’ drug exposure. Bladder cancer with an onset after 6 months’ cumulative exposure to study drug was reported in 4/2187 patients (0.2%) in the placebo group, 3/2216 patients (0.1%) in the empagliflozin 10 mg group, and 7/2190 patients (0.3%) in the empagliflozin 25 mg group (Table 1).

With the number of events being so small, a few additional cases can make a difference to the conclusions drawn. Based on the totality of the data, no imbalance in bladder cancer cases between empagliflozin and placebo were observed in the EMPA-REG OUTCOME trial.

## Abbreviation

SGLT2: Sodium–glucose cotransporter 2
